# Supplementary material for: Agreement between arterial and end-tidal carbon dioxide in adult patients admitted with serious traumatic brain injury
Source: PLoS One. 2024 Feb 2;19(2):e0297113. doi: 10.1371/journal.pone.0297113 (PMC10836696; doi:10.1371/journal.pone.0297113)
Supplement: Attachment — Submitted filename: Response to Reviewers.pdf [file pone.0297113.s001.pdf]

Response to Reviewers: Agreement between arterial and end-tidal carbon dioxide in adult patients admitted with serious traumatic brain injury

Editor

- Thank you for stating the following in the Acknowledgments Section of your manuscript:
  - "This research was supported by the NIHR Cambridge Biomedical Centre (BRC 121520014)"
- We note that you have provided funding information that is not currently declared in your Funding Statement. However, funding information should not appear in the Acknowledgments section or other areas of your manuscript. We will only publish funding information present in the Funding Statement section of the online submission form.
- Please remove any funding-related text from the manuscript and let us know how you would like to update your Funding Statement. Currently, your Funding Statement reads as follows:
  - The authors received no specific funding for this work.
- Please include your amended statements within your cover letter; we will change the online submission form on your behalf

Thank you. The research was supported by data analysts from the NIHR Cambridge Biomedical Centre. The data analysts retrieved the data for no cost.

As instructed, we have removed the funding section of the manuscript. Please could you include the following statement in the online submission form:

"This research was supported by data analysts from the NIHR Cambridge Biomedical Centre (BRC 121520014)."

Reviewer 1

- Would have been helpful if the significance of examining the PaCO2-EtCO2 gradient in patients with TBI could have been more clearly described, such as
  - the rationale for assuming that the PaCO2-EtCO2 gradient is different in patients with TBI than in other patients
  - the clinical significance of the PaCO2-EtCO2 gradient in TBI.

We have made additional reference to the significance of the gradient in patients specifically with TBI (due to correlation of perfusion and CO2) in the background.

- **Abstract**
  - The word "gradient" in the Results section should clearly state what gradient it is

Thank you. The first use of gradient in the results section now reads as '$PaCO_2$-$ETCO_2$ gradient'

- “The gradient was significantly greater in patients with TBI plus concomitant injury, compared to those with isolated TBI”: **Data should be presented.**

Data presented. ‘The gradient was significantly greater in patients with TBI plus concomitant injury, compared to those with isolated TBI (0.9 [0.4-1.5] kPa vs. 0.7 [0.3-1.1] kPa, $p<0.05$).’

- “Patients with TBI who died had a significantly larger gradient compared to those who survived”: **Data should be presented.**

Data presented. ‘Patients with TBI who died (≤30-days) had a significantly larger gradient compared to those who survived (1.2 [0.7-1.9] kPa vs 0.7 [0.3-1.2] kPa, $p<0.005$).’

- **Background**
  - Do you hypothesize that the gradient between $PaCO_2$ and $EtCO_2$ is different between TBI and other patient groups? I would like to know the rationale for why it is necessary to examine the $PaCO_2$-$EtCO_2$ gradient in TBI patients

Thank you. This is an excellent question which related to the rationale of the study and we have added additional comment in the background and explored these concepts further in the discussion.

- **Methods**
  - Are there no data on whether there was spontaneous respiration or mode of ventilation at the time of data collection? The presence or absence of spontaneous breathing and mode of ventilation can have a significant effect on the $PaCO_2$-$EtCO_2$ gradient. In particular, $EtCO_2$ values are highly variable when spontaneous breathing is present

All included patients in this study were intubated and mechanically ventilated. To make this more clear the inclusion criteria now reads as:

‘Patients were included if they were 18 years or older, and sustained a serious or more severe TBI, defined as an Abbreviated Injury Scale (AIS) score ≥3 for ‘head’, who underwent endotracheal intubation either prehospital or in the emergency department, and were admitted to an ICU with ongoing mechanical ventilation.’

- **Results**
  - The authors stated that “The $PaCO_2$ and $ETCO_2$ gradient was significantly greater in patients with a TBI plus concomitant injuries (TBI+) compared to those with isolated-TBI”. However, the difference is very small and the reviewer does not consider this difference to be clinically meaningful (0.9 [0.4-1.5] kPa vs. 0.7 [0.3-1.1] kPa.

Thank you for your comment, we agree that the difference is unlikely to have clinical significance and we explore this further in the discussion, however, we did not feel it appropriate to do so in the presentation of results.

  - Temporal trends: At what point in time the P/F ratio was used to classify the severity of ARDS?

The P/F ratio was extracted at the same times as the $PaCO_2$ measurement. This is included at line 118 and also in Figure 6.

'Additional demographic characteristics, paired $PaCO_2$ and side-stream $ETCO_2$ was extracted from the patient EMR as close as possible to arrival in the ICU, and at 24, 48, 72, 96 and 120 hours after admission (or as near as possible to allow a close temporal link with blood gas data). PaO_2 and FiO_2 were also extracted with the first $PaCO_2$ value to generate PaO_2/FiO_2 (P/F) ratios on admission to the ICU.'

- Association with 30-day mortality: Does Figure 5 show a relationship between gradient and mortality? Please confirm.
    - Given that the PaCO2-EtCO2 gradient is greater in patients with more severe head trauma, it is not surprising that the PaCO2-EtCO2 gradient is greater in patients who died compared to those who survived, which suggests that we are not looking at the relationship between the PaCO2-EtCO2 gradient and mortality, but rather only at the relationship between the severity of head trauma and the PaCO2-EtCO2 gradient

Thank you for your comment, whilst examining the relationship between injury severity and mortality is not within the scope of this project, this is an interesting observation and we have made additional comment in the discussion to reflect this at line 331.

- **Conclusion**
    - Chest injury is not mentioned in either the Methods or the Results, and if you are going to include the following in the Conclusion, you should explain how you analyzed the association between the presence of chest injury and PaCO2-EtCO2: "Amongst adult patients with TBI, the PaCO2-ETCO2 gradient was greater …when associated with concomitant chest injury."

Thank you. This has been changed to 'concomitant injury'.

Reviewer 2

- p4: order of references not correct; 13/14 before 10…

With apologies. The references have now been reviewed and updated manually.

- p4, line 62, a very recent study on this topic could be added (Knapp, J., Doppmann, P., Huber, M. et al. Pre-hospital endotracheal intubation in severe traumatic brain injury: ventilation targets and mortality-a retrospective analysis of 308 patients. Scand J Trauma Resusc Emerg Med 31, 46 (2023). https://doi.org/10.1186/s13049-023-01115-8)

Thank you for drawing our attention to this new relevant paper. This has now been referenced throughout.

- p5 and Results: a collection of AIS groups by organ system would allow a more detailed analysis (adjustment of results). Pre-hospital times? On-scene Time intervals? Concerning the etCO2-PaCO2 gap, haemodynamics plays a central role! Haemodynamics or Lac would, therefore, be significant covariates for a precise analysis. Please go into this in more detail or add it to the limitations. Another reason

for an increased gap could be (too) high tidal volumes; are ventilation parameters available here? (If not, please discuss this and/or include it in the limitations)

- p14. Please describe "concomitant injuries" in more detail here; primarily, thoracic injuries are to be mentioned and not, for example, extremity injuries.

Whilst we agree that prehospital physiological data and timings would provide more of an insight into the temporal changes and the relationship with the gradient, unfortunately these data are not available routinely to the acute hospitals.

Reviewer 3

- The study showed that PaCO2-ETCO2 gradient in TBI + subgroup is larger than that of the isolated -TBI subgroup. Please explain the difference in DISCUSSION

Thank you. This is explored in the paragraph starting at line 291.

- The magnitude of the PaCO2-ETCO2 gradient was associated with increased mortality (Figure 5). Patients who died within 30 days had a larger gradient on admission compared to those who survived; 1.2 [0.7-1.9] kPa and 0.7 [0.3-1.2] kPa respectively, $p<0.005$. Please explain it and provide useful suggestions for this kind of patients (e.g., ventilation management)

Thank you. This is likely due to more severe injury (namely chest) and also VQ mismatch through either primary lung injury or hypovolemia. The following has been added to the discussion at line 239:

"demonstrating the relationship between increased ventilation-perfusion (VQ) mismatch in patients with multi-system injury"

Please explain it and provide useful suggestions for this kind of patients (e.g., ventilation management)

Although this is useful suggestion, it is beyond the scope of this paper to make any recommendations regarding ventilation strategy.
